# I’m trying to read here! How does irrelevant speech affect how you read?

**DOI:** 10.1007/s10339-026-01346-4

**Published:** 2026-05-09

**Authors:** Han Zhang, Kevin F. Miller

**Affiliations:** Department of Psychology, University of Michigan–Ann Arbor, Ann Arbor, USA

**Keywords:** Irrelevant speech, Reading, Eye movements, Distraction

## Abstract

Reading involves a remarkable coordination of perceptual and linguistic processes. This coordination is reflected by a close coupling between reading eye movements and lexical properties of words, such as word frequency and predictability, as well as morpho-syntactic and semantic regularities. Yet, reading is also subject to interference, and a common source of interference is when others are speaking nearby. Then, how does irrelevant speech affect reading as reflected in eye movements? Fifty-nine participants (mean age: 19, SD age: 1.22, 25 females, 34 males, primary language: English) read passages from the PROVO corpus Luke and Christianson (Behav Res Methods 50(2):826–833. 10.3758/s13428-017-0908-4, 2018) under intelligible irrelevant speech or the silence condition, with eye movements recorded. We investigated how irrelevant speech influences the coupling between eye movements and several indicators of lexical difficulty, including word frequency, cloze probability (exact-word predictability), large language model-generated surprisal, morpho-syntactic predictability (part-of-speech and inflection), and semantic similarity (general meaning). Associations between lexical variables and word looking times remained robust during first-pass reading and were unaffected by irrelevant speech, suggesting intact lexical processing during early stages of reading. However, disruption emerged in later eye-movement measures (i.e., total viewing time), where low-frequency and semantically unpredictable words prompted increased re-reading under irrelevant speech. These results indicate that intelligible irrelevant speech selectively interferes with post-lexical, higher-order processing. The increased re-reading of rare and semantically unpredictable words suggests that readers compensate for speech interference by revisiting these words to better integrate their meanings into a coherent understanding of the text.

**Supplementary Information:**

The online version contains supplementary material available at 10.1007/s10339-026-01346-4.

## Introduction

People often find it difficult to read when others are talking nearby. Despite this common experience, researchers have only recently begun to examine how irrelevant speech affects eye movements during reading (Cauchard et al. 2012; Hyönä and Ekholm 2016; Meng et al. 2020, 2025; Vasilev et al. 2019; Yan et al. 2018; Wu et al. 2021; Zang et al. 2024). Understanding how reading unfolds under distraction has both theoretical value for developing ecologically valid models and practical relevance for improving reading performance in real-world settings.

The effect of irrelevant speech on reading can be understood in terms of both the end product of reading and the moment-to-moment reading process. The former is typically assessed by asking participants to answer comprehension questions about texts read under irrelevant speech. A meta-analysis by Vasilev et al. (2018) found a small but reliable detrimental effect of irrelevant speech on comprehension performance (Hedges’ *g* = − 0.26) compared to the silence condition, with the effect being more pronounced for intelligible than unintelligible speech. However, comprehension scores do not capture the impact of irrelevant speech on the moment-to-moment reading process (i.e., *how* people read). Several studies have found that intelligible speech does not impair comprehension accuracy but rather leads to more re-reading (e.g., Cauchard et al. 2012; Hyönä and Ekholm 2016; Yan et al. 2018) and that intelligible speech only impacts comprehension when re-reading is prohibited (Vasilev et al. 2019). Re-reading of earlier portions of the text may compensate for the disruptive effects of irrelevant speech. Local comprehension difficulties may thus be resolved by re-reading parts where difficulties arise (Reichle et al. 2009), and, as a result, speech disruption effects may not be captured by comprehension questions. The current study aims to extend the investigation of *how* people read under irrelevant speech by examining the coupling between eye movements and lexical properties of the text.

Reading comprehension relies on the continuous processing of textual information. This process hinges on a tight coupling between eye movements and lexical properties of words, two of which are word frequency and word predictability. It has been well established that during attentive reading, high-frequency and highly predictable words tend to receive fewer and shorter fixations (Rayner 1998). To understand cognitive processes during reading, researchers commonly distinguish between “early” and “late” measures of eye movements during reading (Clifton et al. 2007; Rayner et al. 1989; Reichle et al. 2010). Early measures include first-fixation duration (the duration of the initial fixation on a word during first-pass reading) and gaze duration (the sum of all fixations on a word before the eyes move past it). Because these measures are restricted to first-pass reading, they are typically taken to index relatively early stages of processing, including lexical access. Substantial research has shown that first-fixation durations are affected by word frequency and word predictability, suggesting that these word properties are processed at the early stages of reading (for reviews, see Clifton et al. 2007; Staub 2015). In contrast, a typical “late” measure is total viewing time (the sum of all fixations on a word), as it includes both initial reading and subsequent re-reading of a word. In line with this distinction, computational models of eye-movement control in reading, such as E-Z Reader, characterize reading as involving both lexical and post-lexical stages (Reichle et al. 2009), with the latter supporting the integration of individual word meanings into a coherent representation of the text. Examining both early and late measures, therefore, provides a useful way to infer the stage of processing at which a given experimental manipulation exerts its effects.

Several studies examining the effects of irrelevant speech on reading eye movements have focused on the global effects of speech, without examining how speech may affect local eye-lexical coupling (Cauchard et al. 2012; Hyönä and Ekholm 2016; Meng et al. 2020, 2025; Wu et al. 2021). Local eye–lexical coupling may be affected both in terms of when disruption emerges (early vs. late measures) and in which direction it manifests (attenuation vs. exaggeration of lexical effects). If irrelevant speech modulates lexical effects during the initial fixation on a word (e.g., first-fixation duration), this would suggest interference with early stages of processing, such as the lexical access of individual words. In contrast, if the effects of irrelevant speech emerge only in subsequent measures beyond the first fixation, this would suggest interference at later stages, such as the integration of individual words into a coherent representation of the text. Changes in the direction of lexical effects are also informative. Attenuation of lexical effects on fixation times would suggest a weakening of the normal coupling between linguistic processing and eye-movement control. Conversely, an exaggeration of lexical effects would indicate that normal processing mechanisms remain engaged but operate less efficiently and/or more effortfully under distraction. Thus, distinguishing early versus late effects, as well as attenuation versus exaggeration of lexical influences, can help identify the mechanisms through which irrelevant speech disrupts reading.

A limited number of studies have examined eye-lexical coupling under irrelevant speech, but these studies have produced mixed results (Vasilev et al. 2019; Yan et al. 2018; Zang et al. 2024). Yan et al. (2018) found that irrelevant speech eliminated the word frequency effect in first-fixation durations when Chinese readers read sentences with a target word of high or low frequency, suggesting that irrelevant speech disrupted early lexical processing. In other words, the eye-lexical coupling may break down at the earliest stages when reading under irrelevant speech. The idea that irrelevant speech attenuates the word frequency effect aligns with the finding that when readers adopt non-comprehension goals, lexical influences on word looking times are reduced, reflecting diminished engagement with lexical processing (e.g., Rayner and Fischer 1996; Reichle et al. 2010). For example, Reichle et al. (2010) reported that word frequency effects on first-pass reading disappeared during self-reported episodes of mind-wandering. If reading eye movements under irrelevant speech exhibits a similar pattern, this would suggest a common mechanism underlying external and internal distractions that disrupts early stages of reading.

Irrelevant speech could also affect word looking times beyond the initial fixation. As noted earlier, recent studies have consistently shown that irrelevant speech increases re-reading (e.g., Cauchard et al. 2012; Hyönä and Ekholm 2016; Vasilev et al. 2019; Yan et al. 2018), a behavior associated with error monitoring and the repair of comprehension difficulties (Reichle et al. 2009). However, it remains unclear whether these re-reading behaviors could exaggerate or attenuate lexical influences on word looking times. One possibility is that irrelevant speech affects only global reading behavior (e.g., overall reading time, re-reading) without exaggerating or attenuating the local eye–lexical coupling. For example, Vasilev et al. (2019) found more re-reading and longer word viewing times under intelligible speech but observed similar word frequency effects in the silence and speech conditions. The authors concluded the opposite of Yan et al. (2018) that intelligible speech did not affect lexical processing during reading. Another possibility is that rather than re-reading all words equally, readers may selectively re-read words with specific lexical characteristics. Familiar or predictable words are likely processed automatically and remain relatively unaffected by speech interference. On the other hand, unfamiliar or unpredictable words require greater processing effort and are more susceptible to comprehension difficulties under irrelevant speech, therefore prompting more re-reading. This would selectively increase total looking times on low-frequency or low-predictability words, thereby exaggerating the observed effects of lexical variables.

Compared to the word frequency effect, even fewer studies have examined how the word predictability effect might be modulated by irrelevant speech. In one study with Chinese readers, Zang et al. (2024) found that the word predictability effect on target words was preserved in both the silence condition and the irrelevant-speech condition, despite the speech condition producing more re-reading. Importantly, readers during naturalistic reading form predictions in a graded rather than all-or-none fashion (Luke and Christianson 2016; Staub 2015). For example, in the sentence “I went to the dog park after eating my ___,” a reader may not know the next word is “dinner”, but they can often anticipate its morpho-syntactic form (a singular noun) or semantic category (something food-related). These partial predictions have been shown to influence eye movements beyond orthographic (exact word) predictability (Luke and Christianson 2016). It is therefore important to examine how multiple aspects of predictive processes might be disrupted by irrelevant speech.

## The current study

Eye movements during reading are usually guided by word frequency and multiple aspects of word predictability (orthographic, morpho-syntactic, and semantic). Previous studies offer an inconclusive picture of how word frequency and word predictability effects are influenced by irrelevant speech in terms of both when disruption emerges (early vs. late measures) and in which direction it manifests (attenuation vs. exaggeration of lexical effects).

The current study sought to conduct a more comprehensive investigation of how lexical effects on word looking times are modulated by intelligible irrelevant speech. Reading materials were drawn from the PROVO corpus (Luke and Christianson 2018), which provides normed measures for: (1) orthographic predictability (cloze probability of the exact word), (2) part-of-speech predictability (e.g., verb or noun), (3) inflectional predictability (e.g., number, tense), and (4) semantic similarity (similarity in meaning).

To complement PROVO’s cloze probabilities, we also computed surprisal values (i.e., the unexpectedness of a word given the context) using a large language model (LLM) as an additional measure of orthographic predictability. Cloze probability scores, traditionally derived from human raters, reflect subjective expectations about the upcoming word. They are closely tied to readers’ comprehension of the preceding text and their world knowledge, but may also be influenced by individual differences, intuitive biases, and characteristics of the sample. In contrast, LLMs are trained on large and diverse text corpora, and their surprisal values reflect statistical regularities present in the training data. As such, they can capture statistical regularities that go beyond immediate human intuitions but may lack the nuanced world knowledge that humans bring to comprehension. Therefore, it is informative to include both measures of word predictability and examine whether their results converge. In the current study, we used a state-of-the-art, open-source LLM, Llama 3.1 (Meta, 2024), to obtain surprisal values of each word.

For each examined lexical variable, we ask

Does irrelevant speech modulate the relationship between eye movements and this lexical variable? If so, at what stage of processing?

A significant interaction between speech condition (silence/speech) and the lexical variable would indicate modulation. The direction of the interaction would indicate whether irrelevant speech exaggerates or attenuates the lexical effect. Early effects of irrelevant speech would be reflected in first-fixation duration and gaze duration, which consider exclusively first-pass reading and therefore index lexical processing. Later effects would be reflected in total viewing time, which includes re-reading and therefore also reflects post-lexical, higher-level comprehension processes.

## Methods

### Participants

Fifty-nine undergraduate students (Mean Age: 19, SD Age: 1.22; 25 Females, 34 Males) participated for course credit. All participants were native English speakers with normal eyesight and hearing. The study’s procedure was approved by the Institutional Review Board at the authors’ university and conforms to US Federal Policy for the Protection of Human Subjects. All participants gave informed consent before participating.

Because this study examined how speech modulates the relationship between multiple lexical variables and eye-movement measures (some of which have not been investigated in this context before), it was difficult to estimate the required sample size a priori. Therefore, we based our sample size on prior studies with similar research questions and designs (Vasilev et al. 2019; Zhang et al. 2018). We targeted a sample size of 50 participants, but ultimately collected data from 59 participants.

### Apparatus and stimuli

#### Reading materials

Reading materials consisted of 55 short passages from the PROVO corpus (Luke and Christianson 2018). The passages were sourced from various daily publications (e.g., news articles, science magazines, and fiction) and had an average length of 50 words (range: 39–62) and 2.5 sentences (range: 1–5).

#### Audio stimuli

Audio distractions consisted of short monologues from the LibriSpeech corpus (http://www.openslr.org/12), a large-scale corpus of English public audiobooks. Audio recordings of two male speakers (speakers 26 and 78) were used. The first speaker read *A Journal of the Plague Year*, and the second speaker read Chaps. 23 and 24 of *Frankenstein*,* or The Modern Prometheus*. The complete recording of each speaker was segmented into multiple short audio files. We selected 80 audio files from each speaker as our pool of audio stimuli. Each audio excerpt lasted 11 to 16 s.

#### Apparatus

Passages were presented on a 20.1-inch screen at approximately 70 cm from the participant. Letters were presented in black Times New Roman font against a white background, with each letter subtending approximately 0.5 degrees of visual angle. Audios were played through a pair of headphones with noise cancellation at approximately 55 decibels. Monocular eye movements were recorded by an Eyelink 1000 System at a sampling rate of 500 Hz under remote tracking mode.

### Procedure

Participants were asked to read each passage for comprehension and to ignore any audio. Participants first completed three practice passages (not from the PROVO corpus but similar in length) to get familiar with the task. On each trial, participants were asked to fixate on a dot appearing at the upper-left corner of the screen. The experimenter, sitting quietly outside the participant’s view, monitored participants’ gaze on a second monitor. Once the experimenter confirmed the participant’s gaze on the fixation dot, the experimenter pressed the space bar to reveal the passage. Participants were asked to fixate on a small dot at the bottom-right corner of the screen after finishing reading. Once the experimenter confirmed participants’ gaze, the experimenter pressed the space bar to end the current trial. The eye tracker was calibrated before the experimental trials began. All 55 passages were displayed in one block. Each trial was randomly determined to have audio playback or not. On audio distraction trials, an audio excerpt was randomly selected from a pool of 160 audio files. Playback started as soon as the passage was shown and looped indefinitely until the trial ended.

#### Data analysis

##### Word frequency information

Word frequency information was obtained from SUBTLEXus, a word frequency norm based on American subtitles (Brysbaert and New 2009). We used the SUBTL_WF_ column from the database, which represents word frequency per million words.

*Surprisal*. We obtained surprisal values using a state-of-the-art, open-source LLM, Llama 3.1 (Meta, 2024). Surprisal is the negative log probability of a given word given its preceding context, with higher surprisal values indicating lower predictability. To compute the surprisal of a given word, we constructed the text sequence from the beginning of the text to the target word, tokenized the sequence into smaller units the model can process, ran these tokens through the model to generate a probability distribution of possible tokens, and computed surprisal for the target word based on this probability distribution. For words consisting of multiple tokens, the individual surprisal values were summed to compute the word-level surprisal.

##### PROVO predictability measures

Cloze probability (“OrthographicMatch” in the PROVO corpus) indicates the proportion of responses that were an orthographic match with the target word. Part-of-speech (POS) predictability (“POSMatch”) indicates the proportion of responses with the same POS as the target word. Inflection predictability (“InflectionMatch”) indicates the proportion of responses with the same inflection as the target word. Semantic match (“LSA_Response_Match_Score”) indicates the extent to which readers could predict the general meaning of the upcoming word. Inflection and semantic match were analyzed for content words (e.g., verbs and nouns) only, as functional words do not have inflection match data and have near-zero semantic match scores in the PROVO corpus.

##### Fixation preprocessing

We defined areas of interest for each word as rectangular boxes surrounding each word (see Figure S1 in the Supplemental Material for an example). Fixations not falling on any words (7.8% of fixations) and those with extreme durations (< 80 or > 2000 msec; 3.83% of fixations) were discarded from analysis. Table 1 lists the definitions of measures analyzed in this study.

**Table 1:** Measures examined in the current study and their definitions

Type	Measure	Definition
Global	Number of Fixations	The total number of fixations on a given text.
Global	Mean Fixation Duration	The mean duration of all fixations on a given text.
Global	Regression Rate	The percentage of fixations re-reading on previous texts.
Global	Skipping Rate	The percentage of words not receiving any fixation.
Local	First-Fixation Duration	The duration of the very first fixation on a word during first-pass reading.
Local	Gaze Duration	The total duration of all first-pass fixations on a word.
Local	Total Viewing Time	The sum of all fixations on a word (including both first-pass and re-reading fixations).

##### Statistical analysis

Statistical analyses were conducted in the R environment (R Core Team 2025) using packages including tidyverse (Wickham et al. 2019), Table 1 (Rich 2025), lmerTest (Kuznetsova et al. 2017), parameters (Lüdecke et al. 2020), patchwork (Pedersen 2025), and bayestestR (Makowski et al. 2019).

Word frequency values ranged from 0.02 to 41857.12 and were *z*-scored to aid model fit. For predictability measures, LLM-generated surprisal values ranged from 0 to 22.38, and PROVO predictability measures (cloze predictability, POS predictability, inflection predictability, and semantic similarity) all ranged from 0 to 1. No transformation was applied to the predictability measures. All models used original word looking time measures, with 0 values set to missing. No other transformations on word looking times were applied.

To examine condition differences on global measures, we constructed linear mixed models with Condition (Silence/Speech, dummy-coded with Silence as the reference level) as a fixed effect. Random effects included by-participant intercepts and slopes as well as by-stimuli intercepts and slopes.

To examine the association between looking times and word properties, we constructed linear mixed models with the specific word property, Condition, and their interaction as fixed effects. Random effects included by-participant intercepts and slopes for all fixed effects as well as by-stimuli intercepts and slopes for all fixed effects. We fit separate models for each combination of word property and looking time measure. Since we have six word properties (word frequency, surprisal, cloze predictability, part-of-speech predictability, inflection predictability, and semantic similarity) and three word looking time measures (first-fixation duration, gaze duration, and total viewing time), we fit a total of 18 linear mixed models examining the association between looking times and word properties. In case of a singular fit, random effects with zero variance were removed. Statistical significance of fixed effects was tested using Satterthwaite approximations. Holm-Bonferroni correction was applied to *p*-values across all models.

Because the current research questions involve evaluating potential null effects (e.g., whether irrelevant speech fails to modulate the word frequency effect), we additionally computed Bayes Factors (BF₀₁) to assess the relative evidence for the null hypothesis compared to the alternative hypothesis. BF₀₁s were computed using Bayesian Information Likelihood (BIC) approximation by comparing a full model against a reduced model that omits the parameter in question (Wagenmakers 2007). Following Jeffreys (1961), values of 1 < BF₀₁ ≤ 3 indicate anecdotal evidence, 3 < BF₀₁ ≤ 10 moderate evidence, 10 < BF₀₁ ≤ 30 strong evidence, 30 < BF₀₁ ≤ 100 very strong evidence, and BF₀₁ > 100 extreme evidence in favor of the null hypothesis.

## Results

### Global measures

As shown in Table 2, when reading under irrelevant speech, participants made significantly more fixations, had longer fixation durations, were more likely to re-read the text, and were less likely to skip words. These results are largely consistent with previous findings (e.g., Cauchard et al. 2012; Vasilev et al. 2019; Yan et al. 2018).

**Table 2:** Means, standard deviations (SDs), and linear mixed model results of global measures

	Mean (SD)		Model Results		
	Silence	Speech	t	*p*	BF_01_
Number of Fixations	44.41 (15.04)	49.65 (18.39)	7.46	< 0.001	< 0.001
Mean Fixation Duration	215.69 (29.67)	222.99 (30.58)	8.40	< 0.001	< 0.001
Regression Rate	12.64% (7.20%)	14.07% (7.39%)	7.00	< 0.001	< 0.001
Skipping Rate	35.69% (11.73%)	33.47% (11.91%)	-6.02	< 0.001	< 0.001

### Word Frequency

**Fig. 1:**
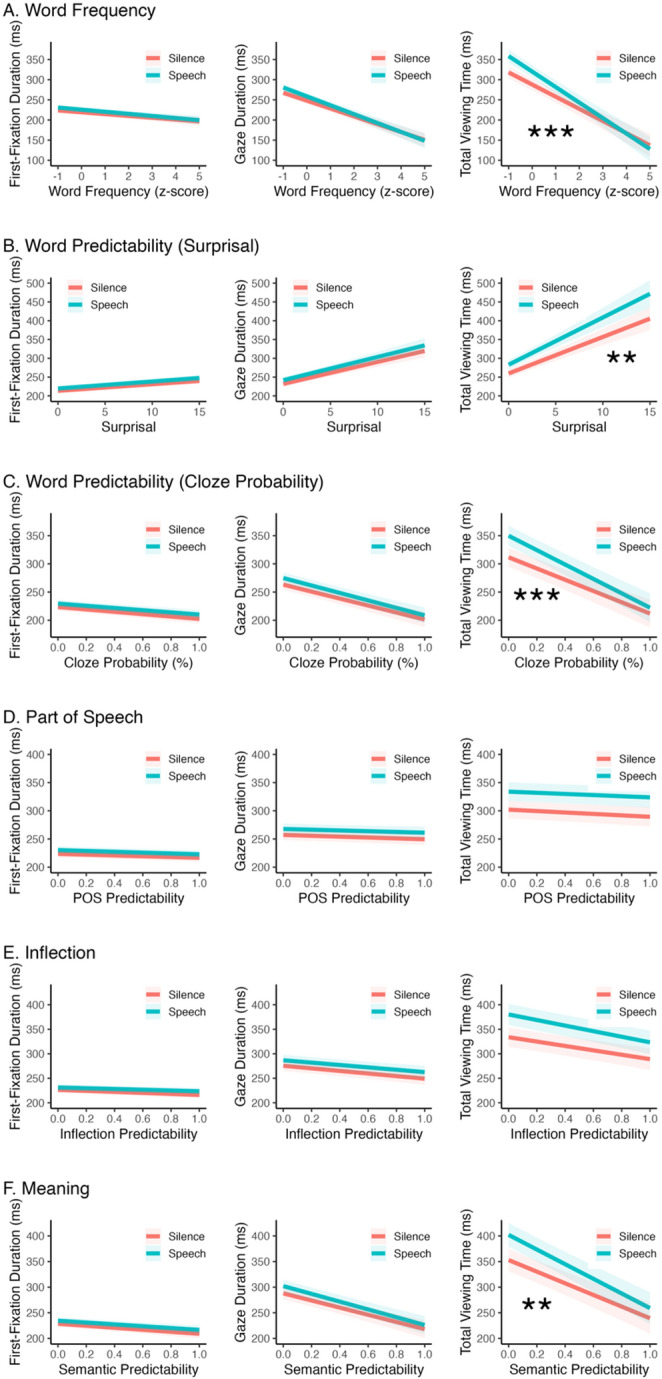
The effects of irrelevant speech on the relationship between lexical variables and looking times. Note. Significant interactions were denoted in asterisks (*** Holm-Bonferroni-adjusted *p* < .001; ** Holm-Bonferroni-adjusted *p* < .01). A complete numerical summary of these results is provided in Tables S1 to S3 of the Supplemental Material

Figure 1 visualizes the effects of irrelevant speech on the relationship between lexical variables and looking times. Here, the results for word frequency are shown in Fig. 1A, with the left panel showing results for first-fixation duration, the middle panel showing results for gaze duration, and the right panel showing results for total viewing time. A complete numerical summary of these results is provided in Table S1 of the Supplemental Material.

Replicating previous work (Rayner 1998), we observed an early effect of word frequency, as indicated by a significant relationship between word frequency and first-fixation durations (Estimate = -4.54, 95% CI [-6.25, -2.82], *t* = -5.26, Holm-Bonferroni adjusted *p* < .001, BF_01_ = 0.001). This means that the very first fixation on a word was shorter if the word had a higher frequency. Additionally, we observed an overall effect of speech on first-fixation duration, such that words read under irrelevant speech received longer initial looking times (Estimate = 6.39, 95% CI [4.66, 8.12], *t* = 7.39, *p* < .001, BF_01_ < 0.001). The effect of word frequency and the effect of irrelevant speech were consistently found for all looking time measures, so we refrain from mentioning them again.

There was no significant interaction between irrelevant speech and word frequency on first-fixation durations (Estimate = -0.66, 95% CI [-2.42, 1.10], *t* = -0.74, *p* > .99, BF_01_ = 223.13) or gaze durations (Estimate = -2.54, 95% CI [-5.19, 0.12], *t* = -1.90, *p* = .790, BF_01_ = 51.57). In both cases, BF_01_s indicate at least very strong evidence in favor of the null hypothesis. However, for total viewing times, there was a significant interaction between word frequency and speech condition (Estimate = -8.30, 95% CI [-11.99, -4.61], *t* = -4.48, *p* < .001, BF_01_ = 0.06). The pattern of this interaction indicates that words with lower frequencies received longer looking times in the speech condition compared to the silence condition. The fact that this effect manifested only in total viewing time and not during first-pass reading (as measured by first-fixation duration and gaze duration) indicates that low-frequency words received additional re-reading in the speech condition.

### Exact-word predictions

Next, we examined how irrelevant speech modulated the associations between exact-word predictability and word looking times. Exact-word predictability was measured using two complementary measures: LLM-generated surprisal values and human-generated cloze predictability scores.

The results of word surprisal are visualized in Fig. 1B. A complete numerical summary is provided in Table S2 of the Supplemental Material. We observed an early effect of surprisal on word looking times, as shown by a significant and positive relationship between surprisal and first-fixation durations (Estimate = 1.76, 95% CI [1.37, 2.16], *t* = 8.83, *p* < .001, BF_01_ < 0.001). Words that were more surprising according to the LLM also received longer looking times on the very first fixation. There was no significant interaction between irrelevant speech and surprisal on first-fixation durations (Estimate = 0.10, 95% CI [-0.29, 0.48], *t* = 0.50, *p* > .99, BF_01_ = 265.47) or gaze durations (Estimate = 0.33, 95% CI [-0.41, 1.08], *t* = 0.89, *p* > .99, BF_01_ = 202.49). In both cases, BF_01_s indicate extreme evidence in favor of the null hypothesis. However, irrelevant speech did significantly modulate the effect of surprisal on total viewing time (Estimate = 2.85, 95% CI [1.31, 4.40], *t* = 3.68, *p* = .009, BF_01_ = 0.678). This pattern indicates that high-surprisal words received additional re-reading under irrelevant speech.

The results were similar for cloze probability, as shown in Fig. 1C. There was an early effect on first-fixation durations, with less predictable words receiving longer first-fixation durations (Estimate = -21.11, 95% CI [-26.64, -15.57], *t* = -7.56, *p* < .001, BF_01_ < 0.001). There was no significant interaction between irrelevant speech and cloze predictability on first-fixation durations (Estimate = 1.61, 95% CI [-3.95, 7.17], *t* = 0.57, *p* > .99, BF_01_ = 251.23) or gaze durations (Estimate = -4.26, 95% CI [-12.02, 3.49], *t* = -1.09, *p* > .99, BF_01_ = 163.10). In both cases, BF_01_s indicate extreme evidence in favor of the null hypothesis. However, we observed a significant interaction effect between cloze probability and irrelevant speech on total viewing times (Estimate = -27.85, 95% CI [-38.16, -17.54], *t* = -5.29, *p* < .001, BF_01_ < 0.001). Together, these results indicate that low predictability words received additional re-reading under irrelevant speech.

### Partial word predictions

Finally, we examined whether irrelevant speech affects the relationship between partial word predictability and word looking times. We examined three aspects of partial word predictability: part-of-speech (POS) predictability, inflection predictability, and semantic similarity. A complete numerical summary of these results is provided in Table S3 of the Supplemental Material.

The results of POS predictability are shown in Fig. 1D. We observed an early effect of POS predictability on first-fixation duration (Estimate = -7.06, 95% CI [-9.91, -4.20], *t* = -4.89, *p* < .001, BF_01_ = 0.005), such that first-fixation durations were shorter for words with a more predictable POS. There was no significant interaction between irrelevant speech and POS in first-fixation duration (Estimate = -0.51, 95% CI [-4.24, 3.22], *t* = -0.27, *p* > .99, BF_01_ = 285.82) or gaze duration (Estimate = 1.09, 95% CI [-4.47, 6.66], *t* = 0.39, *p* > .99, BF_01_ = 274.71), or total viewing time (Estimate = 2.56, 95% CI [-4.78, 9.90], *t* = 0.68, *p* > .99, BF_01_ = 252.88). In these cases, BF_01_s indicate extreme evidence in favor of the null hypothesis.

The results of inflection predictability are shown in Fig. 1E. Inflection predictability also had an early effect on word looking times (Estimate = -10.44, 95% CI [-14.49, -6.39], *t* = -5.07, *p* < .001, BF_01_ = 0.001), such that words with a more predictable inflection received shorter first-fixation durations. There was no significant interaction between irrelevant speech and inflection predictability in first-fixation duration (Estimate = 2.70, 95% CI [-2.65, 8.06], *t* = 0.99, *p* > .99, BF_01_ = 133.65), gaze duration (Estimate = 2.12, 95% CI [-5.91, 10.16], *t* = 0.52, *p* > .99, BF_01_ = 190.97), or total viewing time (Estimate = -12.01, 95% CI [-23.61, -0.40], *t* = -2.04, *p* = .598, BF_01_ = 29.62). In these cases, BF_01_s indicate at least strong evidence in favor of the null hypothesis.

The results of semantic similarity are shown in Fig. 1F. Semantic similarity also had an early effect on looking times (Estimate = -19.73, 95% CI [-26.49, -12.97], *t* = -5.77, *p* < .001, BF_01_ < 0.001), such that first-fixation durations were longer when participants were less likely to predict the upcoming word’s general meaning. There was no significant interaction between irrelevant speech and semantic similarity on first-fixation durations (Estimate = 1.78, 95% CI [-5.35, 8.92], *t* = 0.49, *p* > .99, BF_01_ = 223.86) or gaze durations (Estimate = -6.10, 95% CI [-16.45, 4.25], *t* = -1.16, *p* > .99, BF_01_ = 129.72). In both cases, BF_01_s for both interaction terms indicate extreme evidence in favor of the null hypothesis. However, irrelevant speech did significantly modulate the effect of semantic similarity on total viewing times (Estimate = -29.34, 95% CI [-43.75, -14.93], *t* = -3.99, *p* = .001, BF_01_ = 0.094). This indicates that words with less predictable meaning received additional re-reading under irrelevant speech.

## Discussion

Our results suggest that irrelevant speech did not disrupt lexical processing during early stages of reading: While first-fixation and gaze durations increased overall under speech, the effects of word frequency and predictability remained intact. However, there was clear selective disruption at late processing stages: low-frequency words and low-predictability words showed greater increases in total viewing time in the speech condition, indicating that readers often re-read these words for additional processing. Furthermore, among the various facets of predictability examined, only semantic similarity interacted with irrelevant speech in total viewing time. Morpho-syntactic predictability (part of speech or inflection) did not interact with the speech distraction at any stage of processing.

These results are consistent with previous studies showing that the effects of irrelevant speech typically emerge in later eye-movement measures (e.g., Cauchard et al. 2012; Meng et al. 2020; Vasilev et al. 2019). For example, Meng et al. (2020) asked native Chinese readers to read sentences under three conditions: intelligible speech, unintelligible speech, and silence. One group of participants read the sentences to judge their semantic acceptability, whereas another group read them to detect the presence of a non-character. Intelligible speech disrupted eye movements only in the semantic acceptability task, and these disruptions were confined to relatively late measures, such as total viewing time, due to additional re-reading behavior under the intelligible speech condition compared to the unintelligible speech and silence conditions. The authors concluded that “shallow aspects of perceptual and linguistic processes” are minimally affected by intelligible speech. In contrast, intelligible speech disrupts “higher order processing associated with the formation of a representation of sentential meaning” (p. 1902).

Importantly, the current findings further indicate that re-reading behaviors under speech distraction are selective, focusing on low-frequency and low-predictability words rather than all words equally. Rare and semantically unpredictable words may be especially critical for comprehension because they are unfamiliar and violate readers’ expectations. Thus, it is reasonable that readers selectively re-read these words to “repair” disrupted comprehension.

On the other hand, the current study did not observe the early effects of irrelevant speech reported in previous research (Yan et al. 2018). Specifically, Yan et al. (2018) found that the word frequency effect was delayed under irrelevant speech, such that it did not emerge in first-fixation durations but appeared in gaze duration and total viewing time. A key methodological difference is that Yan et al. (2018) examined the word frequency effect on experimentally controlled target words in short sentences, whereas the present study used a regression-based approach that models word frequency as a continuous predictor across all words in longer paragraphs. As such, their design isolates the effects of word frequency and disruptions from irrelevant speech under tightly controlled conditions, whereas our approach evaluates these relationships as they unfold in a more natural linguistic context. Our approach may reduce sensitivity to subtle early lexical disruptions that require tight experimental control, while being more sensitive to effects that emerge at later, more integrative stages of discourse processing. Future studies could combine the two approaches by examining both all words in a passage and a controlled subset of target words within the same materials (Kliegl et al. 2004).

The current results also contrast sharply with the eye-lexical decoupling observed during mind-wandering that used a similar regression-based approach (e.g., Reichle et al. 2010). Irrelevant speech distraction does not induce this kind of decoupling of eye movements from lexical processing. In our data, readers in the irrelevant speech condition still exhibited robust frequency and predictability effects in early measures, indicating that they were still processing these lexical properties of each word. Thus, our results suggest that irrelevant speech induces a distinct form of distraction: one that leaves early lexical access largely intact but undermines the integration of semantic information, thereby prompting readers to re-read words with low frequency and low semantic similarity.

The current study aimed to elicit clear effects of an ecologically valid and commonly encountered form of distraction on eye movements. Based on this goal, we selected intelligible speech as the distraction stimulus and compared it against a silence condition. However, the present results do not specify which component of intelligible speech drives these effects. According to the phonological-disruption hypothesis, the phonological characteristics of intelligible speech are primarily responsible for interference (Salamé and Baddeley 1982), whereas the semantic-disruption hypothesis attributes interference to its semantic characteristics (Marsh et al. 2008; Martin et al. 1988). Empirical evidence generally supports the latter view, as intelligible speech tends to produce stronger interference effects than unintelligible speech (Vasilev et al. 2018). Although the current study cannot distinguish between these two accounts, the results are consistent with the semantic interference hypothesis: The finding that semantically unpredictable words elicited additional re-reading suggests that the semantic characteristics of irrelevant speech interfered with the semantic integration of the reading material. To fully test this hypothesis, future research should incorporate an unintelligible speech condition to isolate and rule out the potential contribution of phonological characteristics to the observed re-reading patterns.

Overall, our results suggest that intelligible irrelevant speech specifically interferes with post-lexical, higher-order semantic integration rather than lexical or syntactic processing. Increased re-reading of rare and semantically unpredictable words under speech distraction suggests that readers make compensatory efforts to repair disrupted semantic integration. These results provide insights into the mechanisms by which the online reading process is disrupted by irrelevant speech. It also underscores the importance of considering real-world auditory distractions to fully understand the cognitive processes underlying reading comprehension.

## Supplementary Information

Below is the link to the electronic supplementary material.

Supplementary file1 (DOCX 4448 KB)

## Data Availability

The data that support the findings of this study are openly available in the Open Science Framework at https://osf.io/x7paf/.
