# Supplementary material for: I’m trying to read here! How does irrelevant speech affect how you read?
Source: Cogn Process. 2026 May 9;27(3):667–76. doi: 10.1007/s10339-026-01346-4 (PMC13424345; doi:10.1007/s10339-026-01346-4)
Supplement: Supplementary file 1 — Supplementary file1 (DOCX 4448 KB) [file 10339_2026_1346_MOESM1_ESM.docx]

**Supplemental Material**

**Figure S1**

*An example passage with AOIs shown as blue rectangular boxes.*


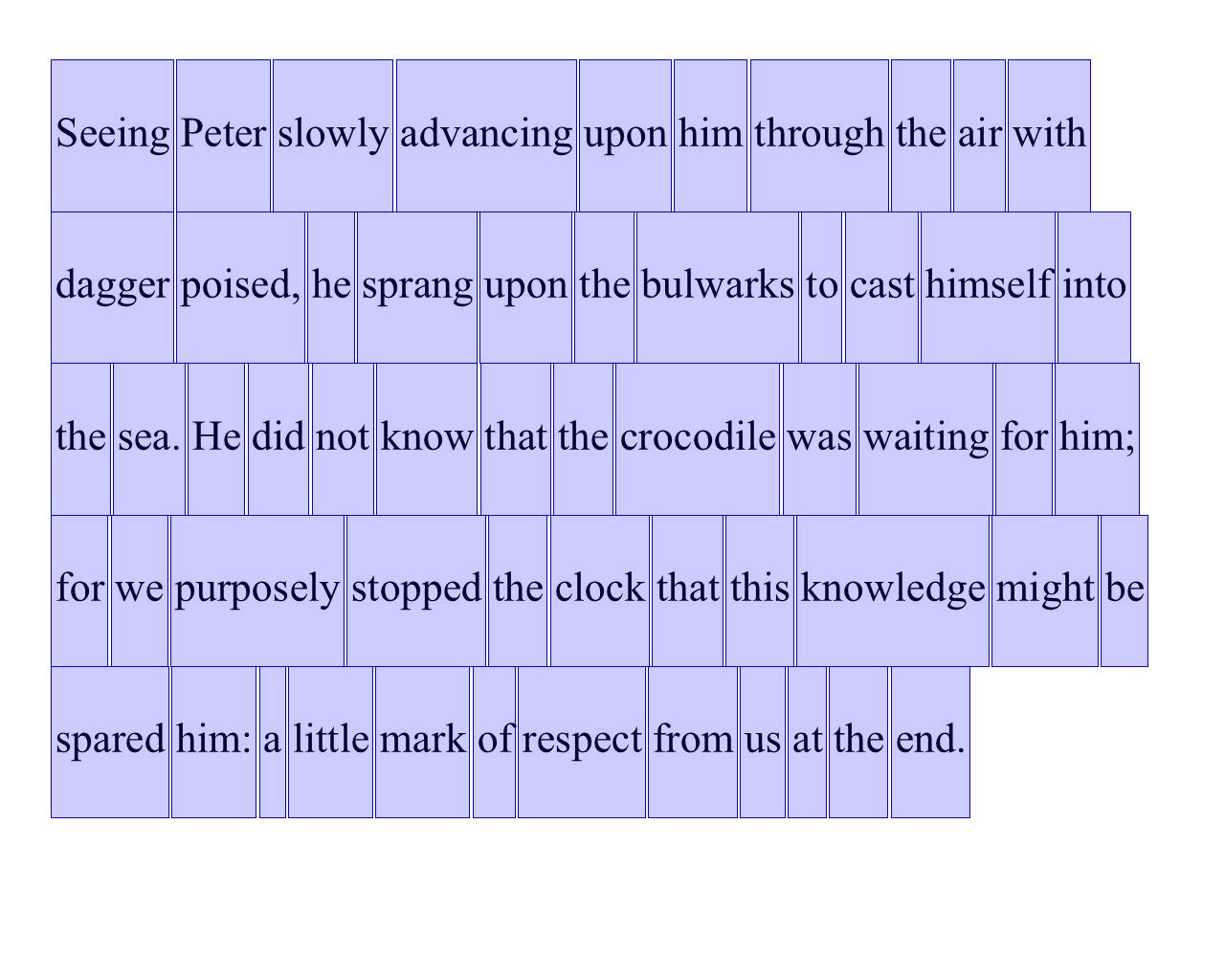


**Table S1**

*Results of linear mixed models examining how irrelevant speech affects the relationship between word frequency and word looking times.*

| Measure | Parameter | Estimate | 95% CI | *t* | *p* | BF_01_ |
| --- | --- | --- | --- | --- | --- | --- |
| First-Fixation Duration | **Frequency** | **-4.54** | **[-6.25, -2.82]** | **-5.26** | **< .001** | **0.001** |
|  | **Condition** | **6.39** | **[4.66, 8.12]** | **7.39** | **< .001** | **< .001** |
|  | Frequency*Condition | -0.66 | [-2.42, 1.1] | -0.74 | > .99 | 223.13 |
|  |  |  |  |  |  |  |
| Gaze Duration | **Frequency** | **-19.52** | **[-22.56, -16.48]** | **-12.75** | **< .001** | **< .001** |
|  | **Condition** | **10.53** | **[7.1, 13.96]** | **6.12** | **< .001** | **< .001** |
|  | Frequency*Condition | -2.54 | [-5.19, 0.12] | -1.9 | 0.790 | 51.57 |
|  |  |  |  |  |  |  |
| Total Viewing Time | **Frequency** | **-30.19** | **[-35.08, -25.3]** | **-12.26** | **< .001** | **< .001** |
|  | **Condition** | **32.21** | **[24.7, 39.73]** | **8.54** | **< .001** | **< .001** |
|  | **Frequency*Condition** | **-8.3** | **[-11.99, -4.61]** | **-4.48** | **< .001** | **0.06** |

*Note.* significant effects (*p* < .05) are highlighted in bold font. *p*-values are corrected using the Holm-Bonferroni method.

**Table S2**

*Results of linear mixed models examining how irrelevant speech affects the relationship between exact-word predictability (surprisal and cloze predictability) and word looking times.*

| Measure | Parameter | Estimate | 95% CI | *t* | *p* | BF_01_ |
| --- | --- | --- | --- | --- | --- | --- |
| *LLM-generated Surprisal* | | | | | | |
| First-Fixation Duration | **Surprisal** | **1.76** | **[1.37, 2.16]** | **8.83** | **< .001** | **< .001** |
|  | **Condition** | **6.14** | **[3.88, 8.4]** | **5.36** | **< .001** | **< .001** |
|  | Surprisal*Condition | 0.1 | [-0.29, 0.48] | 0.5 | > .99 | 265.47 |
|  |  |  |  |  |  |  |
| Gaze Duration | **Surprisal** | **5.84** | **[4.74, 6.95]** | **10.48** | **< .001** | **< .001** |
|  | **Condition** | **9.81** | **[6.08, 13.53]** | **5.23** | **< .001** | **0.002** |
|  | Surprisal*Condition | 0.33 | [-0.41, 1.08] | 0.89 | > .99 | 202.49 |
|  |  |  |  |  |  |  |
| Total Viewing Time | **Surprisal** | **9.72** | **[7.78, 11.66]** | **9.91** | **< .001** | **< .001** |
|  | **Condition** | **23.27** | **[16.86, 29.69]** | **7.21** | **< .001** | **< .001** |
|  | **Surprisal*Condition** | **2.85** | **[1.31, 4.4]** | **3.68** | **0.009** | **0.678** |
|  |  |  |  |  |  |  |
| *Cloze Predictability* | | | | | | |
| First-Fixation Duration | **Cloze Predictability** | **-21.11** | **[-26.64, -15.57]** | **-7.56** | **< .001** | **< .001** |
|  | **Condition** | **6.12** | **[4.26, 7.99]** | **6.53** | **< .001** | **< .001** |
|  | Cloze Predictability *Condition | 1.61 | [-3.95, 7.17] | 0.57 | > .99 | 251.23 |
|  |  |  |  |  |  |  |
| Gaze Duration | **Cloze Predictability** | **-61.87** | **[-72.6, -51.13]** | **-11.43** | **< .001** | **< .001** |
|  | **Condition** | **11.65** | **[7.99, 15.31]** | **6.33** | **< .001** | **< .001** |
|  | Cloze Predictability *Condition | -4.26 | [-12.02, 3.49] | -1.09 | > .99 | 163.10 |
|  |  |  |  |  |  |  |
| Total Viewing Time | **Cloze Predictability** | **-99.81** | **[-118.75, -80.86]** | **-10.44** | **< .001** | **< .001** |
|  | **Condition** | **38.17** | **[30.76, 45.58]** | **10.24** | **< .001** | **< .001** |
|  | **Cloze Predictability *Condition** | **-27.85** | **[-38.16, -17.54]** | **-5.29** | **< .001** | **< .001** |

*Note.* Significant effects (*p* < .05) are highlighted in bold font. *p*-values are corrected using the Holm-Bonferroni method.

**Table S3**

*Results of linear mixed models examining how irrelevant speech affects the relationship between partial word predictability (part-of-speech, inflection, and semantic content) and word looking times.*

| Model | Parameter | Estimate | 95% CI | *t* | *p* | *BF_01_* |
| --- | --- | --- | --- | --- | --- | --- |
| *Part-of-Speech (POS) Predictability* | | | | | | |
| First-Fixation Duration | **POS Predictability** | **-7.06** | **[-9.91, -4.20]** | **-4.89** | **< .001** | **0.005** |
|  | **Condition** | **6.71** | **[4.15, 9.28]** | **5.15** | **< .001** | **< .001** |
|  | POS Predictability*Condition | -0.51 | [-4.24, 3.22] | -0.27 | > .99 | 285.82 |
|  |  |  |  |  |  |  |
| Gaze Duration | POS Predictability | -7.67 | [-13.81, -1.52] | -2.48 | 0.23 | 11.15 |
|  | **Condition** | **10.54** | **[6.16, 14.92]** | **4.75** | **< .001** | **0.008** |
|  | POS Predictability*Condition | 1.09 | [-4.47, 6.66] | 0.39 | > .99 | 274.71 |
|  |  |  |  |  |  |  |
| Total Viewing Time | POS Predictability | -12.7 | [-21.91, -3.49] | -2.74 | 0.127 | 8.78 |
|  | **Condition** | **31.9** | **[23.88, 39.91]** | **7.88** | **< .001** | **< .001** |
|  | POS Predictability*Condition | 2.56 | [-4.78, 9.90] | 0.68 | > .99 | 252.88 |
|  |  |  |  |  |  |  |
| *Inflection Predictability* | | | | | | |
| First-Fixation Duration | **Inflection Predictability** | **-10.44** | **[-14.49, -6.39]** | **-5.07** | **< .001** | **0.001** |
|  | Condition | 4.76 | [1.02, 8.50] | 2.50 | 0.204 | 9.82 |
|  | Inflection Predictability*Condition | 2.70 | [-2.65, 8.06] | 0.99 | > .99 | 133.65 |
|  |  |  |  |  |  |  |
| Gaze Duration | **Inflection Predictability** | **-26.32** | **[-35.29, -17.36]** | **-5.84** | **< .001** | **< .001** |
|  | **Condition** | **11.22** | **[4.67, 17.77]** | **3.37** | **0.02** | **0.873** |
|  | Inflection Predictability*Condition | 2.12 | [-5.91, 10.16] | 0.52 | > .99 | 190.97 |
|  |  |  |  |  |  |  |
| Total Viewing Time | **Inflection Predictability** | **-44.8** | **[-58.68, -30.92]** | **-6.41** | **< .001** | **< .001** |
|  | **Condition** | **46.32** | **[34.73, 57.91]** | **7.9** | **< .001** | **< .001** |
|  | Inflection Predictability*Condition | -12.01 | [-23.61, -0.40] | -2.04 | 0.598 | 29.62 |
|  |  |  |  |  |  |  |
| *Semantic Similarity* | | | | | | |
| First-Fixation Duration | **Semantic Similarity** | **-19.73** | **[-26.49, -12.97]** | **-5.77** | **< .001** | **< .001** |
|  | **Condition** | **5.89** | **[2.95, 8.84]** | **3.94** | **0.002** | **0.129** |
|  | Semantic Similarity*Condition | 1.78 | [-5.35, 8.92] | 0.49 | > .99 | 223.86 |
|  |  |  |  |  |  |  |
| Gaze Duration | **Semantic Similarity** | **-69.84** | **[-84.12, -55.55]** | **-9.69** | **< .001** | **< .001** |
|  | **Condition** | **14.13** | **[8.40, 19.87]** | **4.86** | **< .001** | **0.004** |
|  | Semantic Similarity*Condition | -6.1 | [-16.45, 4.25] | -1.16 | > .99 | 129.72 |
|  |  |  |  |  |  |  |
| Total Viewing Time | **Semantic Similarity** | **-113.63** | **[-136.57, -90.69]** | **-9.82** | **< .001** | **< .001** |
|  | **Condition** | **49.04** | **[38.78, 59.31]** | **9.45** | **< .001** | **< .001** |
|  | **Semantic Similarity*Condition** | **-29.34** | **[-43.75, -14.93]** | **-3.99** | **.001** | **0.094** |

*Note.* Significant effects (*p* < .05) are highlighted in bold font. *p*-values are corrected using the Holm-Bonferroni method.
